# Efficacy and safety of lanthanum carbonate on chronic kidney disease–mineral and bone disorder in dialysis patients: a systematic review

**DOI:** 10.1186/1471-2369-14-226

**Published:** 2013-10-17

**Authors:** Chenglong Zhang, Ji Wen, Zi Li, Junming Fan

**Affiliations:** 1 Department of nephrology, West China Hospital of Sichuan University, Chengdu, China; 2 Luzhou Medical College, Luzhou, China

**Keywords:** Lanthanum carbonate, Chronic kidney disease mineral and bone disorder, Hemodialysis, Peritoneal dialysis, Systematic review

## Abstract

**Background:**

Chronic kidney disease–mineral and bone disorder (CKD–MBD) is a common complication in CKD patients, particularly in those with end-stage renal disease that requires dialysis. Lanthanum carbonate (LC) is a potent, non-aluminum, non-calcium phosphate binder. This systematic review evaluates the efficacy and safety of LC in CKD-MBD treatment for maintenance-dialysis patients.

**Methods:**

A systematic review and meta-analysis on randomized controlled trials (RCTs) and quasi-RCTs was performed to assess the efficacy and safety of LC in maintenance hemodialysis or peritoneal dialysis patients. Analysis was performed using the statistical software Review Manager 5.1.

**Results:**

Sixteen RCTs involving 3789 patients were identified and retained for this review. No statistical difference was found in all-cause mortality. The limited number of trials was insufficient to show the superiority of LC over other treatments in lowering vascular calcification or cardiovascular events and in improving bone morphology, bone metabolism, or bone turn-over parameters. LC decreased the serum phosphorus level and calcium × phosphate product (Ca × P) as compared to placebo. LC, calcium carbonate (CC), and sevelamer hydrochloride (SH) were comparable in terms of controlling the serum phosphorus, Ca × P product, and intact parathyroid hormone (iPTH) levels. However, LC resulted in a lower serum calcium level and a higher bone-specific alkaline phosphatase level compared with CC. LC had higher total cholesterol and low-density lipoprotein (LDL) cholesterol levels compared with SH. LC-treated patients appeared to have a higher rate of vomiting and lower risk of hypercalcemia, diarrhea, intradialytic hypotension, cramps or myalgia, and abdominal pain. Meta-analysis showed no significant difference in the incidence of other side effects. Accumulation of LC in blood and bone was below toxic levels.

**Conclusions:**

LC has high efficacy in lowering serum phosphorus and iPTH levels without increasing the serum calcium. Current evidence does not show a higher rate of adverse effects for LC compared with other treatments, except for a higher incidence of vomiting. Moreover, LC accumulation in blood and bone was below toxic levels. Well-designed studies should be conducted to evaluate the long-term effects of LC.

## Background

With the progression of renal failure, patients frequently have disorders in bone and mineral metabolism [1]. This group of disorders is collectively called chronic kidney disease–mineral and bone disorder (CKD–MBD) and includes pathogenically linked biochemical abnormalities, bone diseases, and cardiovascular (CV) and soft tissue calcification [1]. Gradual decline in renal phosphorus clearance during CKD progression leads to hyperphosphatemia [2], which is a key factor in the development of MBD. Approximately 40% of dialysis patients reportedly suffer from hyperphosphatemia [3]. Increasing evidence shows that hyperphosphatemia promotes CV calcification [4] and is an important predictor of mortality in end-stage renal disease (ESRD) patients undergoing dialysis [5-7]. Thus, lowering the serum phosphorus levels is a promising therapeutic goal.

Serum phosphorus levels in ESRD patients can be controlled by dietary restrictions, adequate dialysis schedule, and oral phosphate binders. A number of effective phosphate binders are currently available. For several years, aluminum- and calcium-based salts were widely used as phosphate binders because of their high efficacy and low price. However, aluminum has well-documented toxic effects that can lead to osteomalacia or encephalopathy [8,9], whereas large doses of calcium-based salts can contribute to CV calcification [10]. The use of aluminum- and calcium-free phosphate binders may address these issues. Sevelamer hydrochloride (SH) is another useful phosphate binder for ESRD patients [11,12]. However, its use is limited by the associated metabolic acidosis and gastrointestinal disorders as well as by the high dosage required to achieve adequate phosphate control [11-14].

Lanthanum carbonate (LC) is another phosphate binder that does not contain aluminum or calcium. Lanthanum is a naturally occurring “rare-earth” element that has a phosphate-binding capacity similar to that of aluminum. However, it is poorly absorbed in the human intestine and has an absolute oral bioavailability of only 0.00089% [15,16]. Early studies in dialysis patients with ESRD demonstrated the effects of LC in lowering phosphorus levels during short-term follow-up compared with a placebo and calcium carbonate (CC) [17,18]. Recently published studies observed the efficacy of LC in controlling the phosphorus level, reducing CV calcification [19], and in altering bone morphology [20-22] during a longer follow-up [21-24].

A previously published systematic review evaluated the efficacy and safety of LC in CKD patients and mainly focused on biochemical parameters [25]. We conducted a systematic review of the efficacy and safety of LC in ESRD patients undergoing dialysis, particularly in terms of long-term outcomes such as mortality, CV calcification, and bone disorder. The results were then compared with those of a placebo or other phosphate binders.

## Methods

### Search strategy

Randomized controlled trials (RCTs) or quasi-RCTs (in which allocation to treatment was obtained by alternation, alternate medical records, date of birth, or other predictable methods) of LC in patients with hemodialysis (HD) or peritoneal dialysis (PD) were searched in MEDLINE, EMBASE, the Cochrane Renal Group Specialised Register, and the Cochrane Central Register of Controlled Trials (CENTRAL) using the following criteria without any language restrictions: “lanthanum carbonate OR Fosrenol AND (dialysis OR hemodialysis OR peritoneal dialysis OR end stage renal disease”. Animal or pediatric studies (with subjects below 18 years of age) were excluded without further review. Two authors independently screened the titles and abstracts of the remaining studies and discarded studies that were not applicable. However, studies and reviews that possibly include relevant data or information were initially retained. The latest date for the search was March 31, 2013.

### Studies

All RCTs and quasi-RCTs that investigated the safety and effectiveness of LC in maintenance-HD or PD patients were considered eligible for inclusion.

### Participants

ESRD patients who regularly receive HD or PD, aged ≥ 18 years old, and did not use LC previously (at least > 1 week) were included in this study. Patients with any of the following conditions were excluded: 

1. Pregnancy or lactation. 

2. Significant hypercalcemia [serum calcium > 11.0 mg/dL (2.75 mmol/L)] or hypocalcemia [serum calcium < 7.9 124 mg/dL (1.98 mmol/L)].

3. Significant gastrointestinal disorders such as active peptic ulcer, ulcerative colitis and Crohn’s disease, intestinal obstruction, or fecal impaction.

4. Malignancy.

5. Any exposure to other investigational drugs within 30 days prior to the start of the study.

### Interventions

Intervention included the use of LC on ESRD patients receiving HD or PD regardless of dosage, mode of administration, or duration of treatment. The comparisons were as follows:

1. LC + routine treatment versus placebo + routine treatment.

2. LC + routine treatment versus calcium-based binders (CBBs) + routine treatment.

3. LC + routine treatment versus SH + routine treatment.

4. LC + routine treatment versus other non-calcium binders (NCBs) or previous phosphate binders + routine treatment.

Routine treatment: HD or PD and supportive treatment. Supportive treatment included methods that treat underlying kidney or medical diseases or improve other disorders linked to kidney failure, such as anemia and hypertension. Other medications for CKD-MBD treatment, such as calcitriol and calcimimetics, could be used when needed, but the use of such medications should be applied parallelly both in the treatment group and the control group. Dietary restriction was not mandatory. Routine treatments in the LC group and the control group should be comparable.

### Outcome measures

#### Primary outcomes

1. All-cause mortality.

2. Cardiovascular events.

Cardiovascular events were defined as fatal or nonfatal myocardial infarction, fatal or nonfatal cerebrovascular event (stroke), or the development of coronary artery disease.

#### Secondary outcomes

1. Vessel calcification (VC), including those of the aorta, coronary artery, and cardiac valves, as determined by spiral computed tomography.

2. Biochemical outcomes such as levels of serum phosphorus, serum calcium, calcium × phosphate product (Ca × P), intact parathyroid hormone (iPTH), 1,25-(OH)D_3_, 25-(OH)_2_D_3_, total alkaline phosphatase (TAP), bone-specific alkaline phosphatase (BAP), and blood lipid.

3. Bone disorder (including bone morphology and bone metabolism).

4. Lanthanum contents in bone, liver, and blood.

5. Inflammatory biomarker such as C-reactive protein (CRP).

6. Side effects of medications.

### Quality assessment

The quality of included studies was independently assessed by two authors who were not blind to authorship or journal of publication. The check list designed by the Cochrane Renal Group was used. Disagreements were resolved by consulting with an independent third party. The quality items assessed were the allocation concealment, blinding, intention-to-treat analysis, and completeness of follow-up. Blinding was assessed for investigators, participants, outcome assessors, and data analysts.

### Data extraction and management

Data extraction was independently performed by two authors using standard data-extraction forms. When more than one publication of one study existed, reports were grouped together, and the most recent or most complete dataset was used. For studies that only displayed the results within diagrams from which data could not be retrieved, e-mails were sent to the authors to request for accessible data.

### Statistical analysis

Analyses were performed using Review Manager 5.1. The results of dichotomous outcomes (all-cause mortality and cardiovascular events) were expressed as risk ratios (RR) with 95% confidence intervals (CI). The mean difference (MD) was obtained when continuous scales of measurement were used to assess the treatment effects (e.g., serum calcium, Ca × P, and iPTH), whereas the standardized mean difference (SMD) was obtained when different scales were used. Between-study heterogeneity was assessed using the chi-square test. Random-effects analysis was used when *I*^*2*^ > 50%, whereas fixed-effects analysis was used when *I*^*2*^ < 50% [26].

## Results

### Search results

**Figure 1:**
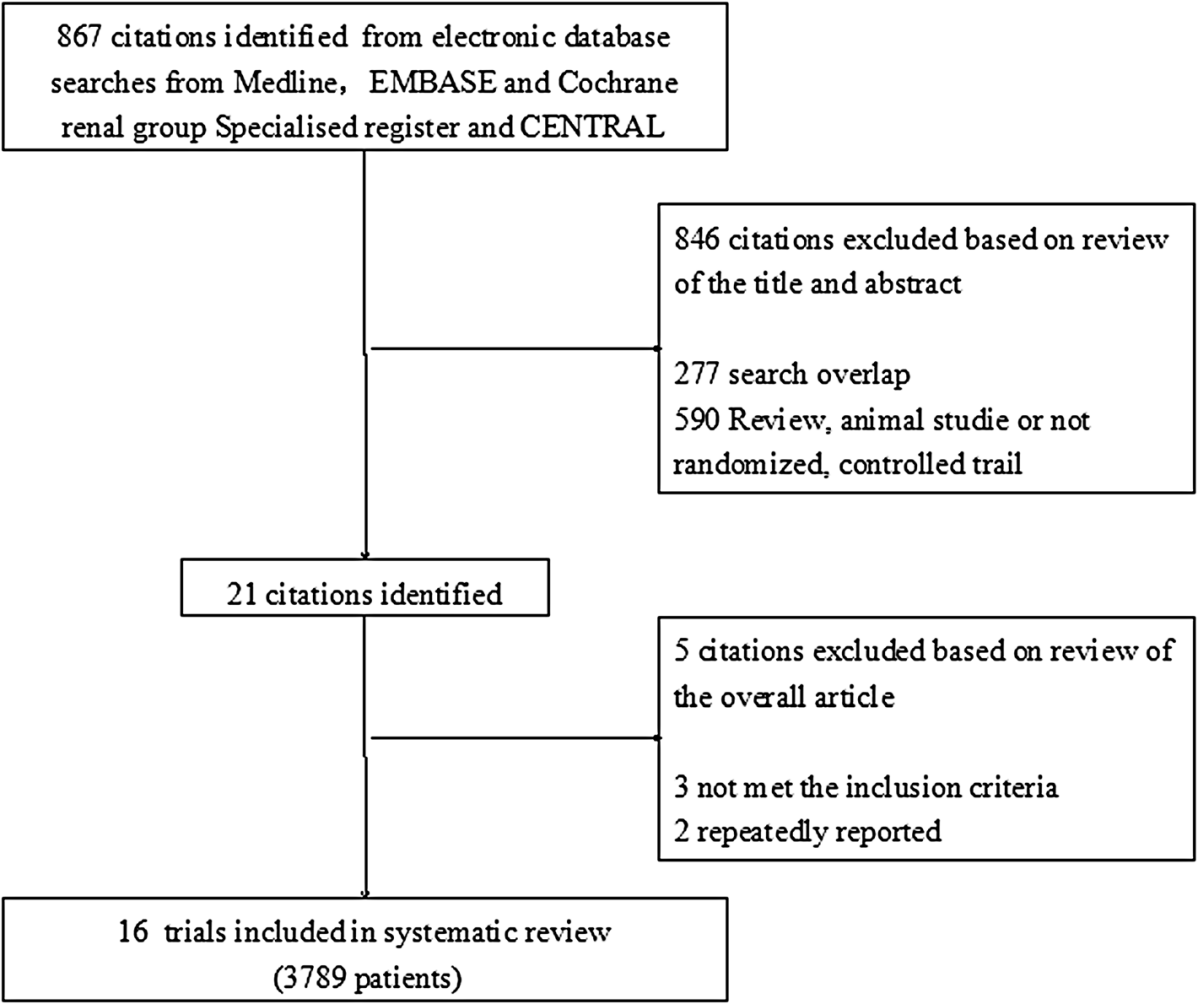
Procedure used for the trial selection.

Literature search identified 867 articles, 846 of which did not involve RCTs or quasi-RCTs and were thus excluded. Animal studies were also excluded. The full texts of 21 articles were analyzed, and an additional 3 were excluded because none of them met the inclusion criteria [27-29]. After excluding 2 repeatedly published studies [24,30], 16 articles [17-22,31-40] were identified and retained for this review. The 16 studies involved 3789 patients, 2100 of which were in the LC groups and 1689 in the control groups (241 in the placebo group, 534 in the CBB group, 205 in the SH group, and 709 in the NCB group) (Figure 1).

### Characteristics of the included studies

The characteristics of the included studies are summarized in Table 1. Fourteen studies were prospective RCTs, whereas two were randomized crossover studies. All the included studies were published in English. The two crossover studies [32,33] combined the data of phase 1 and phase 2 (before and after exchange of treatment) together to do the comparison but did not report the data for each phase. Three studies included HD and CAPD patients [20,37,39]. Eleven studies included only HD patients. One study included CAPD patients [39]. One study did not mention what kind of the dialysis method was used in the included patients [22]. Sample size ranged from 24 to 1359, and all but two studies had more than 500 participants [23,31].

**Table 1:** **Characteristics of trials of LC for CKD**-**MBD in dialysis patients**

**Study**	**Intervention**		**No. of patients**	**Duration (Week)**	**Dialysis methods**
	**LC group**	**Control group**			
**Fouad Al-Baaj 2005**	LC 375-2250 mg/d	Placebo	36	4	HD
**Melanie S. Joy 2003**	LC 375 mg, 750 mg, 1500 mg, 2250 mg, 3000 mg/day	Placebo	93	4	HD and CAPD
**Finn WF 2006**	LC<3000 mg/d (serum phosphate ≤5.9 mg/dl)	pre-study phosphate binder (serum phosphate ≤5.9 mg/dl)	1359	104	HD
**Patrick. C 2003**	LC<3750 mg/d	CC<9000 mg/d	98	52	HD
**N. D TOUSSAINT 2011**	LC (serum phosphate in the normal range)	CC (serum phosphate in the normal range)	45	72	HD and CAPD
**T.shigematsu 2008**	LC+CC-liked placebo 750, 1500, 2250 mg/d (serum phosphate at 3.5-5.5 mg/dl)	CC+LC-liked placebo 1500, 3000, 4500 mg/d(serum phosphate 3.5-5.5 mg/dl)	258	8	HD
**H H Mallache 2008**	LC<3000mg/d (serum phosphate≤5.9mg/dl)	Previous phosphate binder (serum phosphate ≤5.9 mg/dl)	65	52	HD
**T.shigematsu 2007**	LC 750 mg, 1500 mg, 2250 mg, 3000 mg/day	Placebo	142	6	HD
**Spasovski GB 2006**	LC<3000 mg/d (serum phosphate <1.8 mmol/L)	CC< 4000 mg/d (serum phosphate <1.8 mmol/L)	24	52	Not describe
**A.J. Hutchison 2005**	LC 250-3000 mg/d	CC 1000-9000 mg/d	767	25	HD
**S.-S. Chiang 2005**	LC 375-3000 mg/d (can’t change during study)	Placebo	61	4	HD
**Finn WF 2004**	LC 225 mg, 675 mg, 1350 mg, 2250 mg/day	Placebo	144	6	HD
**Yong Kyu Lee 2013**	LC 1500 mg at start, regulate to control the serum phosphate at 3.5-5.5 mg/dl	CC 3000 mg at start, regulate to control the serum phosphate at 3.5-5.5 mg/dl	50	24	CAPD
**Xu 2013**	LC 1500 mg-3000 mg/day	Placebo	230	4	HD and CAPD
**Sprague S.M 2009**	LC 3,000 mg/day	SH 6,400 mg/day	333	4	HD
**Kasai S 2012**	LC 375–2250 mg/day	SH 750–9000 mg/day	84	13	HD

The follow-up period ranged from 4 weeks to 3 years. One study [21] observed 33 patients in the LC group and 32 patients in the standard therapy group for 1 year and 32 and 24 patients for 2 years, respectively. Another study [22] followed up on 12 patients in the LC group and 12 in the CC group after 1 and 3 years, respectively. Only the data for the first year were complete and were extracted for meta-analysis. In summary, the follow-up periods of 9 studies were less than 24 weeks [17,32-38,40], those of 4 studies ranged from 24 weeks to 1 year [20,21,31,40], and those of 3 studies were longer than 1 year [19,22,23].

In terms of intervention, 6 studies compared LC with a placebo [17,34,36-38,40], 6 compared LC with CBBs [19,20,22,31,34,39], 2 compared LC with SH [32,33], and 2 compared LC with previous phosphate binders [21,23].

Four studies [17,20,21,32] used calcitriol in the routine treatment, whereas one study [17] used calcimimetics. The baseline of the usage of above medication were parallel between the LC group and the control group in all of the studies.

### Study quality

A summary of the quality measurements is shown in Table 2. All 16 studies performed random allocation, but only 2 [19,34] described the use of randomization. A considerable part of the studies included in this review were not blinded. Only 8 studies [17,32,34-38,40] reported blinding of patients and physicians, and 1 [19] reported blinding of outcome assessors. Intention-to-treat analysis was performed in 10 of the 16 studies, and fulfillment of follow-up was high in most of the studies except for two, which had long follow-up durations.

**Table 2:** Summary of quality measures of included studies

	**Randomisation method**	**Allocation concealment**	**Blinding: Participants**	**Blinding: Investigators**	**Blinding: Outcome assessors**	**Blinding: Data assessors**	**ITT**	**% Follow-up**
**Fouad Al-Baaj 2005**	**NS**	**NS**	**Yes**	**Yes**	**No**	**No**	**No**	**94**
**Melanie S. Joy 2003**	**NS**	**NS**	**Yes**	**Yes**	**No**	**No**	**Yes**	**87**
**Finn WF 2006**	**NS**	**NS**	**No**	**No**	**No**	**No**	**No**	**38**
**Patrick.C 2003**	**NS**	**NS**	**No**	**No**	**No**	**No**	**Yes**	**64**
**N. D TOUSSAINT 2011**	**Computer-generated random numbers**	**Yes**	**No**	**No**	**No**	**Yes**	**Yes**	**67**
**T.shigematsu 2008**	**NS**	**NS**	**Yes**	**Yes**	**No**	**No**	**Yes**	**99**
**H H Mallache 2008**	**NS**	**NS**	**No**	**No**	**No**	**No**	**No**	**47**
**T.shigematsu 2007**	**A single stream scheme**	**Yes**	**Yes**	**Yes**	**No**	**No**	**No**	**91**
**Spasovski GB 2006**	**NS**	**NS**	**NS**	**NS**	**NS**	**NS**	**No**	**83**
**A.J. Hutchison 2005**	**NS**	**NS**	**No**	**No**	**No**	**No**	**Yes**	**58**
**S.-S. Chiang 2005**	**NS**	**NS**	**Yes**	**Yes**	**No**	**No**	**Yes**	**69**
**Finn WF 2004**	**NS**	**NS**	**Yes**	**Yes**	**No**	**No**	**Yes**	**63**
**Yong Kyu Lee 2013**	**NS**	**NS**	**NS**	**NS**	**NS**	**NS**	**No**	**69**
**Xu 2013**	**NS**	**NS**	**Yes**	**Yes**	**NS**	**NS**	**Yes**	**99**
**Sprague, S.M 2009**	**NS**	**NS**	**Yes**	**Yes**	**NS**	**NS**	**Yes**	**90**
**Kasai, S 2012**	**NS**	**NS**	**NS**	**NS**	**NS**	**NS**	**Yes**	**95**

*NS*: Not Stated.

### Outcome

#### Effect on all-cause mortality

Only two studies [19,23] reported all-cause mortality of patients using LC and other phosphate binders. No significant difference was observed between the LC and the control in the risk of lowering all-cause mortality (2 studies, 1404 patients, RR: 0.85, 95% CI: 0.69 to 1.04).

#### Effect on cardiovascular events

Only one study [19] reported incidences of cardiovascular events. In the study, 3 in 22 LC-treated patients and 4 in 23 CC-treated patients experienced at least one cardiovascular event. No significant difference was observed between the LC and CBB groups in terms of the risk of lowering cardiovascular events (1 study, 45 patients, RR: 0.78, 95% CI: 0.20 to 3.11).

#### Effect on vessel calcification

One study [19] reported an improvement in aortic vascular calcification. In the study, patients were randomized to either LC (*n*=22) or CC (*n*=23). Patients in the LC group showed significantly less aortic VC progression than those in the CC group (difference from baseline −99.6 HU, 95% CI: –150.5 to −48.8, *p* < 0.001). None of the trials reported calcification of the coronary artery or cardiac valves.

#### Effects on biochemical outcomes

**Figure 2:**
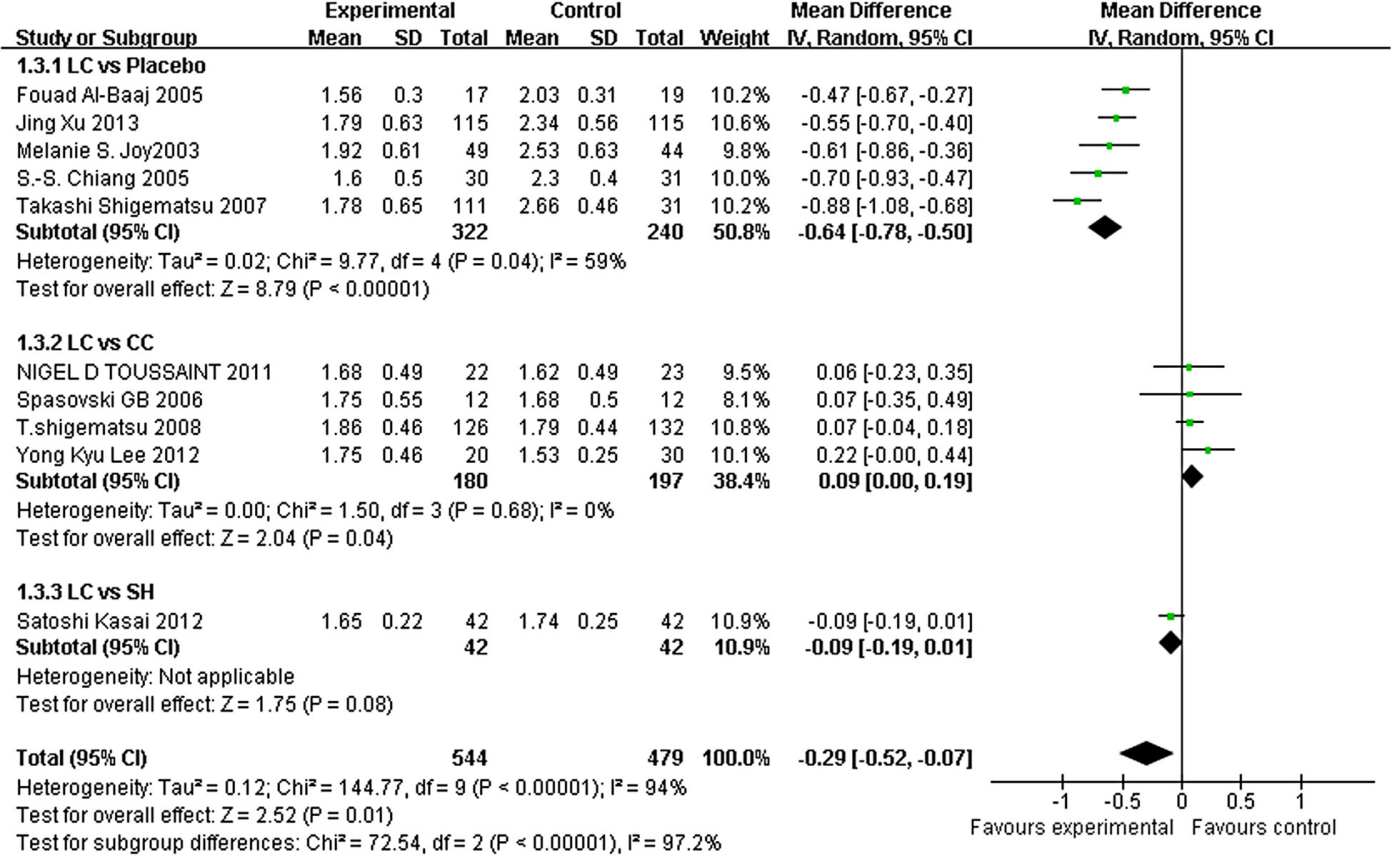
**Forest plot of serum phosphate level of patients treated with LC and control therapy.** Studies were identified by name of the first author and year of publication. Mean differences (MDs) were pooled using the random-effect model and shown on a scale of −1 to 1.

Fourteen studies [19-22,31-40] compared the serum phosphorus level after treatment with LC with that of a control. Six studies reported the results in diagrams only and did not provide definite figures. The figures for two studies were eventually acquired by writing the authors [34,35]. The remaining four studies were not included because the authors did not respond [20,21,23,32]. The serum phosphorus levels were compared in 10 other studies, 5 of which compared LC with a placebo [34,36-38,40], 4 compared LC with CC [19,22,35,39], and 1 compared LC with SH [33]. Meta-analysis showed that LC significantly lowered the serum phosphorus level compared with the placebo (5 studies, 562 patients, MD: –0.64, 95% CI: –0.78 to −0.50), whereas no difference was observed between the LC and CC groups (4 studies, 377 patients, MD: 0.09, 95% CI: 0.00 to 0.19) and between the LC and SH groups (1 study, 84 patients, MD: –0.09, 95% CI: –0.19 to 0.01) (Figure 2).

**Figure 3:**
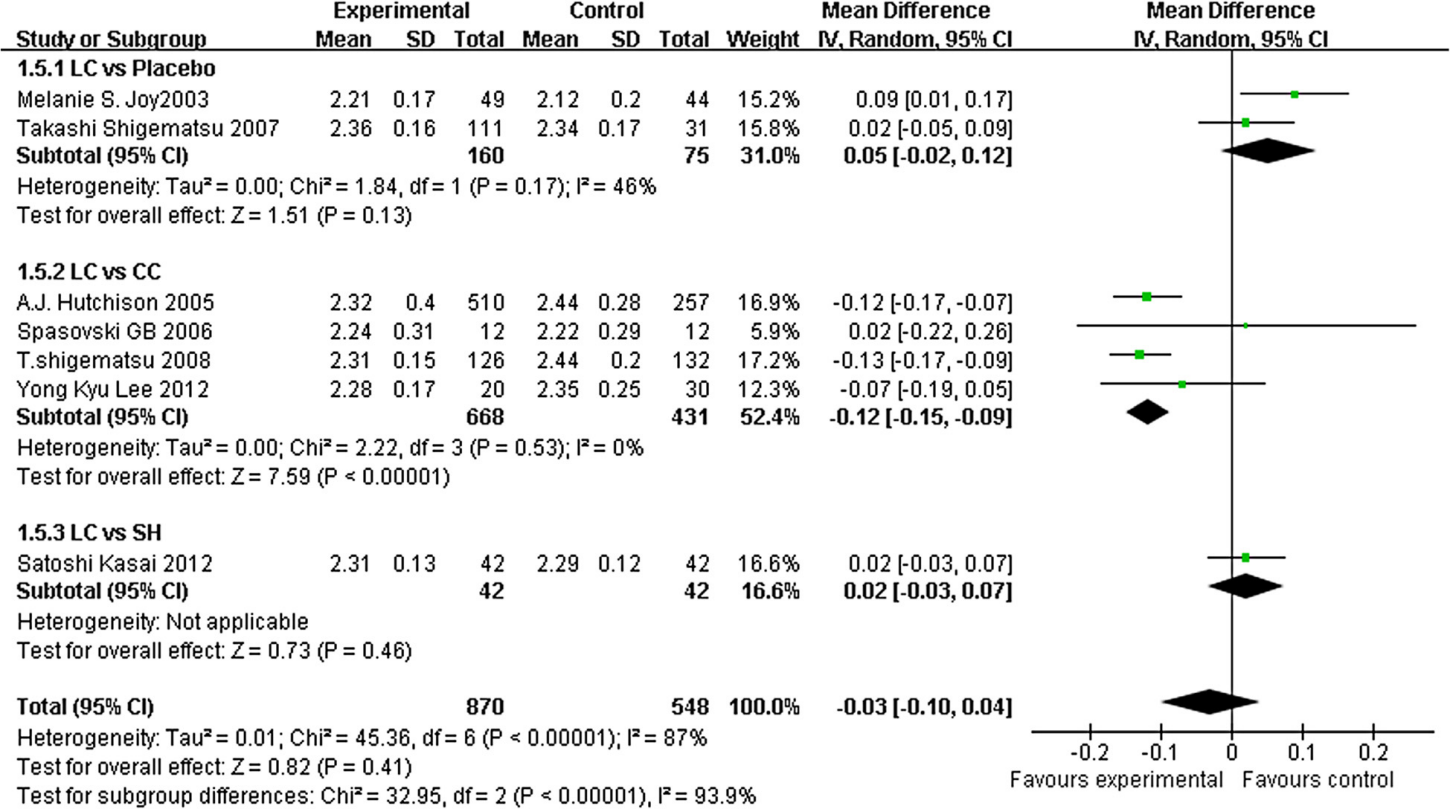
**Forest plot of serum calcium in patients treated with LC and control therapy.** Studies were identified by name of the first author and year of publication. Mean differences (MDs) were pooled using the random-effect model and shown on a scale of −0.2 to 0.2.

Seven studies [22,31,33-35,37,39] provided reports on serum calcium levels. Analysis of their results showed no difference between LC and the placebo (2 studies, 235 patients, MD: 0.05, 95% CI: –0.02 to 0.12) or between LC and SH (1 study, 84 patients, MD: 0.02, 95% CI −0.03 to 0.07). CC-treated patients had higher calcium levels than those treated with LC (4 studies, 1099 patients, MD: –0.12, 95% CI: –0.15 to −0.09) (Figure 3).

**Figure 4:**
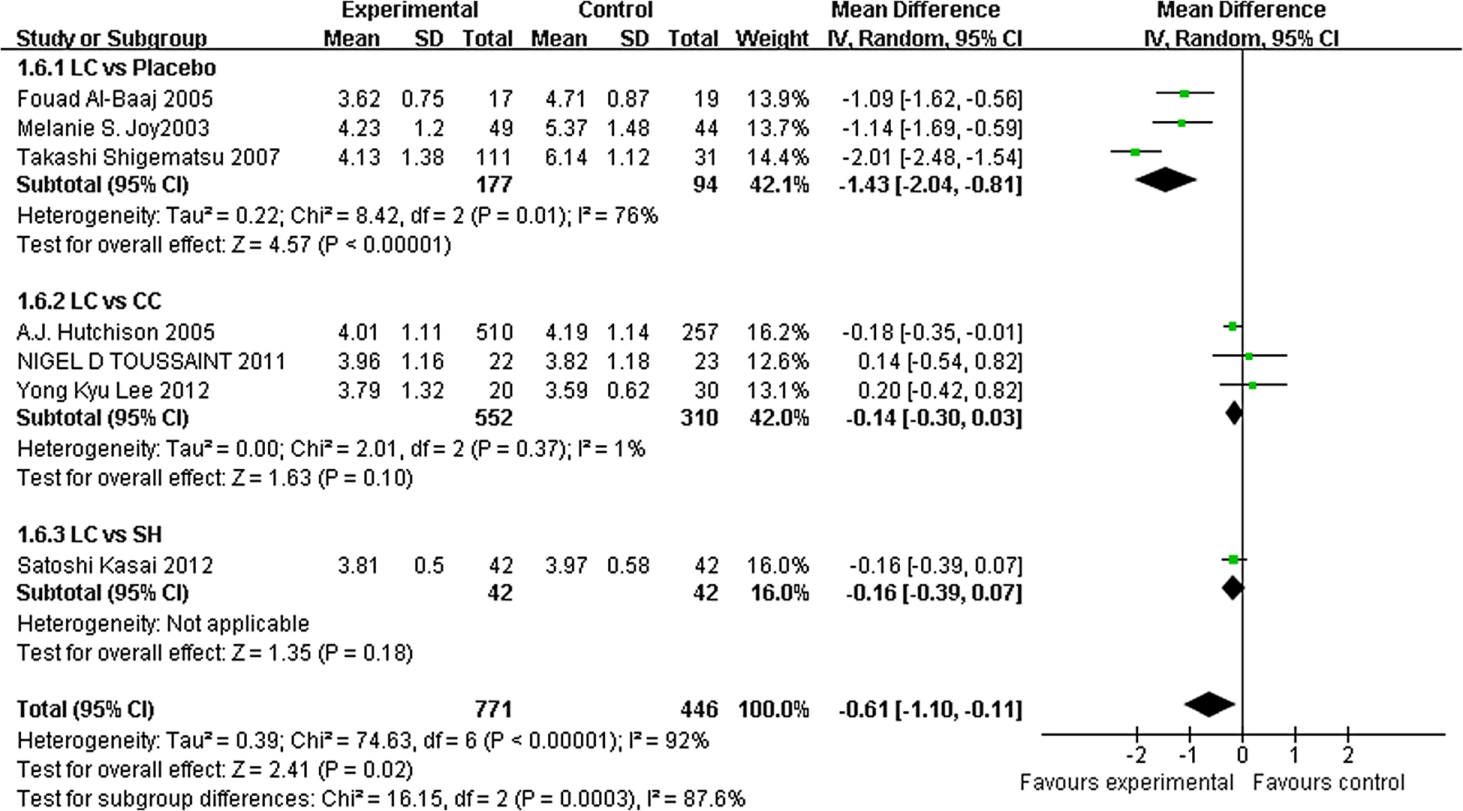
**Forest plot of Ca × P product in patients treated with LC and control therapy.** Studies were identified by name of the first author and year of publication. Mean differences (MDs) were pooled using the random-effect model and shown on a scale of −2 to 2.

Seven studies [19,31,33,34,36,37,39] reported Calcium × Phosphate Product levels and showed that patients treated with LC had lower Ca × P than those treated with a placebo (3 studies, 271 patients, MD: –1.43, 95% CI: –2.04 to −0.81). By contrast, no significant difference was observed between LC and CC (3 studies, 862 patients, MD: –0.14, 95% CI: –0.30 to 0.03) and between LC and SH (1 study, 84 patients, MD: –0.16, 95% CI −0.39 to 0.07) (Figure 4).

**Figure 5:**
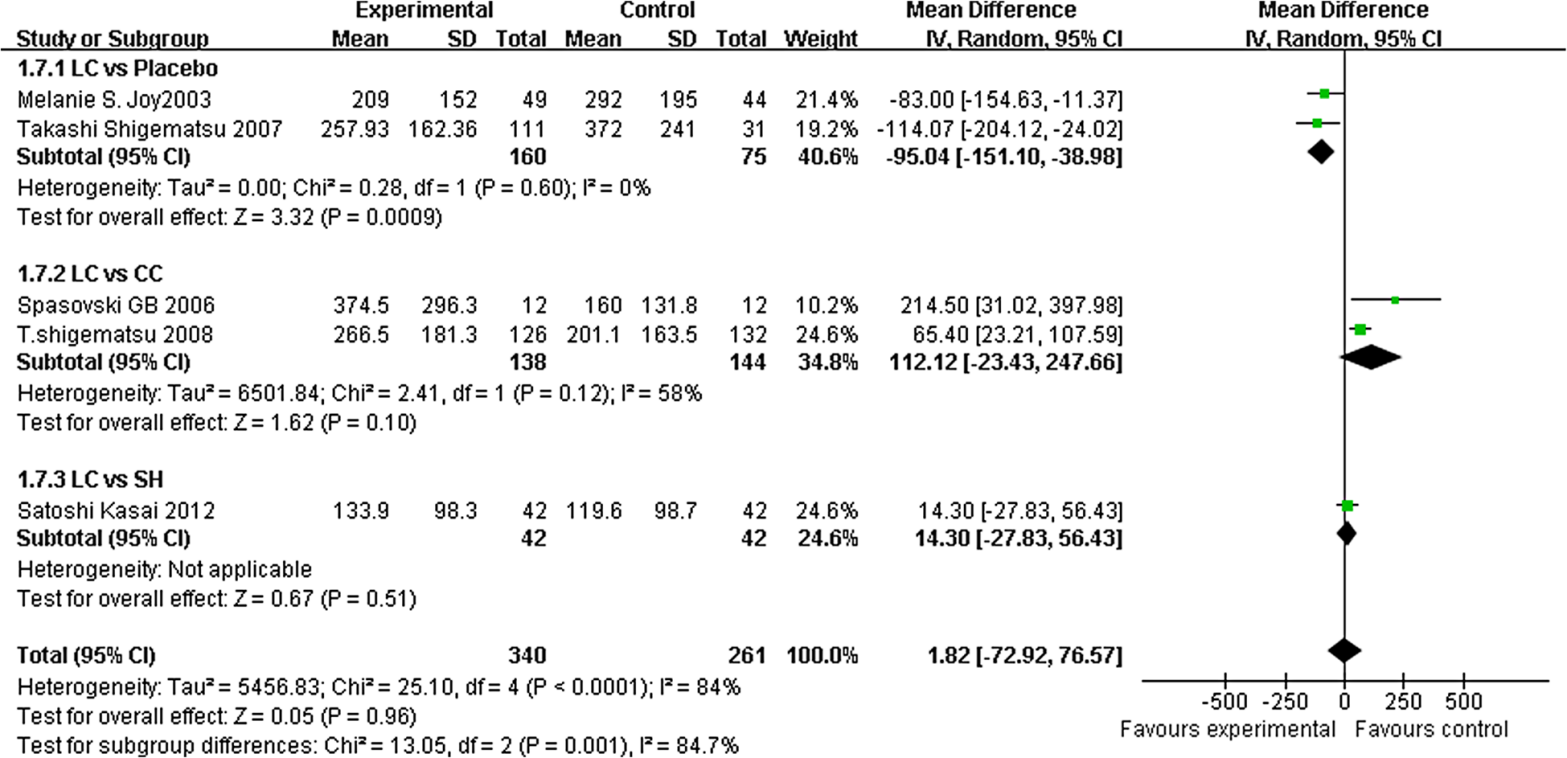
**Forest plot of iPTH in patients treated with LC and control therapy.** Studies were identified by name of the first author and year of publication. Mean differences (MDs) were pooled using the random-effect model and shown on a scale of −500 to 500.

Four studies [22,33-35,37] reported the change in iPTH levels and showed that LC-treated patients achieved lower iPTH levels than those treated with placebos (2 studies, 235 patients, MD: –95.04, 95% CI: –151.10 to −38.98). By contrast, no significant differences were observed between LC and CC (2 studies, 282 patients, MD: 112.12, 95% CI: –23.43 to 247.66) and between LC and SH (1 study, 84 patients, MD: 14.30, 95% CI: –27.83 to 56.43) (Figure 5).

Spasovski et al. [22] followed up the patients at 1 or 3 years after treatment and showed no difference between the 1,25-(OH)D_3_ levels of the LC and CC groups (1 study, 24 patients, MD: –0.80, 95% CI: –27.16 to 25.56). In a similar manner, no difference was observed between the 25-(OH)_2_D_3_ levels (1 study, 24 patients, MD: 11.00, 95% CI: –6.21, to 28.21) and TAP (1 study, 24 patients, MD: 7.80, 95% CI: –31.73 to 47.33) of the LC and CC groups. One study compared LC with SH and showed no difference in TAP (1 study, 84 patients, MD: 7.00, 95% CI: –55.03 to 69.03). Three studies [22,23,31] reported the BAP levels; patients in the LC group had higher BAP than those who continued to use the previous phosphate binder (1 study, 1359 patients, MD: 4.90, 95%: CI 2.73 to 7.07). By contrast, no difference was found between the BAP levels of LC and CC (1 study, 24 patients, MD: 2.50, 95% CI: –10.44 to 15.44) and between LC and SH (1 study, 84 patients, MD: 1.30, 95% CI: –6.55 to 9.15).

The results also showed that when compared with the SH group, the LC group had higher levels of total cholesterol (1 study, 84 patients, MD: 25.00, 95% CI: 12.17 to 37.83) and LDL cholesterol (1 study, 84 patients, MD: 20.00, 95% CI: 10.16 to 29.84) [33]. Other studies did not investigate the difference between the lipid levels of the LC and control groups (placebo, CC, or previous phosphate binders).

### Bone disorder

Three studies [20-22] involved patients who received at least one bone biopsy. However, these studies used different parameters that a meta-analysis was not possible. D’Haese et al. [20] performed bone biopsy in 33 patients in the LC group and 30 patients in the CC group at the baseline and one year after treatment, respectively. The subtypes of bone diseases were similarly distributed in both groups at the baseline. In the LC group, the number of patients with renal osteodystrophy (ROD) decreased from 12 (36%) at the baseline to 6 in the 1-year follow-up (18%), whereas that in the calcium group increased from 13 (43%) to 16 (53%). One patient in the LC group and 6 in the CC group evolved toward adynamic bone.

Malluche et al. [21] performed bone biopsy in 32 patients in the LC group and 33 patients in the CC group at the baseline and 1 and 2 years after treatment. Their results showed that under similar phosphorus control, the LC group showed improvements in the bone turnover and bone volume; the improvements were particularly significant in the 1-year and 2-year groups, respectively. No significant change in the bone turnover or bone volume was observed in the standard phosphate-binder therapy group. Spasovski et al. [22] followed up on patients 1 or 3 years after treatment and did not observe any differences in the osteoblast numbers and mineral apposition rates between the LC and CC groups.

### Lanthanum contents in bone, liver, and blood

Seven studies [20,22,23,31,34,37,38] measured the plasma or serum lanthanum level. Most of the results showed slightly increased lanthanum levels in the blood of the LC groups but did not indicate any statistical difference, except in one study [22], which reported significantly higher plasma lanthanum levels in the LC group compared with that in the CC group. No study reported lanthanum contents exceeding the limit of quantification, a condition that would be harmful to the body. Three studies [20-22] detected the lanthanum content in the bone and showed no significant difference in the bone lanthanum contents between LC and control group. Meta-analysis was not performed because the unit for lanthanum content varied among the studies. No study reported the lanthanum content in the liver or other organs.

### Inflammatory biomarker

None of trials reported any inflammatory biomarker such as CRP.

### Side effects of medications

Only 4 studies [19-21,39] did not evaluate the side effects of medications. Results showed that LC had a lower risk of diarrhea when compared with placebo (4 studies, 395 patients, RR 0.31, 95% CI 0.15 to 0.65). When compared with CBBs, there was a higher rate of vomiting (2 studies, 1058 patients, RR 1.51, 95% CI 1.08 to 2.12) and a lower rate of hypercalcaemia (5 studies, 1220 patients, RR 0.12, 95% CI 0.04 to 0.38) in patient treated with LC. Our meta-analysis also showed that when compared with previous phosphate binders, there was a lower rates of intradialytic hypotension (1 study, 1359 patients, RR: 0.66, 95% CI: 0.53 to 0.82), cramps or myalgia (1 study, 1359 patients, RR: 0.76, 95% CI: 0.63 to 0.92), and abdominal pain (1 study, 1359 patients, RR: 0.73, 95% CI: 0.59 to 0.91) in LC-treated patient. No significant differences were found in the incidences of nausea, constipation, bronchitis, dyspepsia, rhinitis, pruritus, and dialysis complications (Table 3).

**Table 3:** **Side**-**effects of medications**

	**Fixed-effects model**		**Random-effects model**		**Heterogeneity**	
	**RR (95% CI)**	**P value**	**RR (95% CI)**	**P value**	**P value**	**I**^**2**^** (%)**
**Vomiting**	1.06 [0.91, 1.23]	0.45	1.22 [0.81, 1.84]	0.33	0.04	54%
LC vs Placebo	2.35 [0.95, 5.80]	0.06	1.87 [0.55, 6.37]	0.32	0.22	32%
LC vs CBBs	1.51 [1.08, 2.12]	0.02	1.51 [1.08, 2.11]	0.02	0.37	0%
LC vs previous phosphate binders	0.90 [0.76, 1.06]	0.20	0.90 [0.76, 1.06]	0.20	-	-
**Diarrhea**	0.79 [0.68, 0.93]	0.003	0.69 [0.40, 1.18]	0.17	0.001	70%
LC vs Placebo	0.31 [0.15, 0.65]	0.002	0.29 [0.14, 0.62]	0.001	0.53	0%
LC vs CBBs	1.29 [0.84, 1.98]	0.24	1.29 [0.84, 1.98]	0.24	-	-
LC vs previous phosphate binders	0.75 [0.63, 0.90]	0.001	0.75 [0.63, 0.90]	0.001	-	-
**Nausea**	1.03 [0.91, 1.17]	0.61	1.25 [0.85, 1.84]	0.26	0.06	52%
LC vs Placebo	2.06 [0.82, 5.16]	0.12	1.60 [0.49, 5.16]	0.43	0.26	27%
LC vs CBBs	1.42 [1.01, 2.01]	0.05	1.80 [0.70, 4.64]	0.22	0.09	66%
LC vs previous phosphate binders	0.93 [0.81, 1.07]	0.32	0.93 [0.81, 1.07]	0.32	-	-
**Constipation**	0.72 [0.46, 1.12]	0.14	0.70 [0.36, 1.35]	0.29	0.23	31%
**Hypercalcaemia**	0.10 [0.06, 0.16]	<0.00001	0.12 [0.04, 0.38]	0.0002	0.009	71%
LC vs CBBs						
**Intradialytichypotension**	0.70 [0.58, 0.85]	0.0004	0.72 [0.54, 0.96]	0.03	0.28	21%
LC vs Placebo	3.10 [0.34, 28.17]	0.31	3.10 [0.34, 28.17]	0.31	-	-
LC vs CBBs	0.83 [0.51, 1.36]	0.47	0.83 [0.51, 1.36]	0.47	-	-
LC vs previous phosphate binders	0.66 [0.53, 0.82]	0.0002	0.66 [0.53, 0.82]	0.0002	-	-
**Cramps or Myalgia**	0.81 [0.68, 0.97]	0.02	0.83 [0.65, 1.07]	0.15	0.32	13%
LC vs Placebo	1.29 [0.38, 4.35]	0.68	1.29 [0.38, 4.35]	0.68	-	-
LC vs CBBs	1.12 [0.64, 1.95]	0.69	1.12 [0.64, 1.95]	0.69	-	-
LC vs NCBs	0.76 [0.63, 0.92]	0.005	0.76 [0.63, 0.92]	0.005	-	-
**Abdominal pain**	0.75 [0.61, 0.92]	0.006	0.74 [0.60, 0.91]	0.004	0.64	0%
LC vs Placebo	2.14 [0.40, 11.44]	0.37	1.93 [0.35, 10.55]	0.45	0.56	0%
LC vs CBBs	0.60 [0.18, 2.00]	0.40	0.60 [0.18, 2.00]	0.40	-	-
LC vs previous phosphate binders	0.73 [0.59, 0.91]	0.004	0.73 [0.59, 0.91]	0.004	-	-
**Bronchitis**	0.82 [0.66, 1.03]	0.08	0.82 [0.66, 1.03]	0.08	0.97	0%
**Dyspepsia**	0.24 [0.09, 0.61]	0.003	0.21 [0.04, 1.17]	0.007	0.13	52%
LC vs Placebo						
**Rhinitis**	1.20 [0.69, 2.08]	0.52	1.20 [0.69, 2.08]	0.52	0.64	0%
**Pruritus**	4.04 [0.56, 29.27]	0.17	3.84 [0.48, 30.48]	0.20	0.55	0%
LC vs Placebo						
**Dialysis complication**	0.68 [0.39, 1.17]	0.16	0.67 [0.38, 1.16]	0.15	0.39	0%

## Discussion

A comprehensive search for RCTs was performed to evaluate the efficacy and safety profile of lanthanum in maintenance-dialysis patients. A total of 16 trials involving 3789 patients met our criteria and were enrolled in our meta-analysis. Our results show no lanthanum-induced decrease in all-cause mortality or cardiovascular events. Only one RCT [19] reported on vascular calcification and showed that lanthanum delayed the progression of aortic calcification compared with CC. Lanthanum efficiently lowered the serum phosphorus, Ca × P, and iPTH levels compared with placebos. Moreover, lanthanum showed equal efficiency in lowering serum phosphorus, Ca × P, and iPTH levels as calcium bicarbonate but with a lower serum calcium level. No statistical differences in 1,25-(OH)D_3_, 25-(OH)_2_D_3_ and TAP were observed between lanthanum and CC. However, lanthanum caused a statistically significant increase in the BAP level compared to previous phosphate binder. No differences were observed between SH and LC in controlling serum phosphorus, serum calcium, TAP, and BAP levels. However, SH reduced the total cholesterol and the LDL cholesterol levels. The efficacy of lanthanum on bone disorder was reported in only a few studies, and different parameters were used. Thus, our meta-analysis cannot draw reliable conclusions.

The two trials that observed all-cause mortality reported no difference in the risks of all-cause mortality between lanthanum and calcium bicarbonate [19] or standard therapy (without lanthanum) [23]. Wilson et al. [24] performed a trial involving 1354 patients and conducted follow-up examinations for 40 months. The study contributed 98.9% of the weight in our all-cause mortality analysis because of its large sample size. The study found no significant difference between the overall mortality rates of the LC treatment [19.9% (135/680)] and standard therapy [23.3% (157/674)]. Subgroup analysis showed that the mortality for patients aged > 65 years was significantly lower in the LC treatment than in the standard therapy. This trend is highly similar to that of the Dialysis Clinical Outcomes Revisited (DCOR) study [41], which is the largest randomized comparator-controlled trial that assessed the mortality risks of non-calcium-based binders (sevelamer) and CC.

Vascular calcification is a common and severe problem associated with mortality in adult ESRD patients [42]. LC was demonstrated to attenuate the progression of vascular calcification in several animal models [43,44]. In the present analysis, only one trial [19] observed this outcome and reported that compared with CC, lanthanum carbonate was associated with the reduced progression of aortic calcification in 30 HD patients for over 18 months. Additional clinical studies involving large sample sizes and long-term follow-up must be conducted to determine whether lanthanum confers the advantage of inhibiting vascular calcification to dialysis patients. A prospective, large-scale observational study [Study of Hyperphosphatemia in CKD5D Patients Undergoing Hemodialysis (STOP-HD trial): UMIN-ID 000002002] is currently underway to confirm the inhibitory effect of LC on vascular calcification. The results of this study are being anticipated.

A small number of trials performed bone biopsy; however, the efficiency on bone disorder was difficult to evaluate. Two trials [20,21] found improvements in renal osteodystrophy in lanthanum-treated patients compared with those treated with CC or with their previous phosphate binders (without lanthanum). However, another trial [22] showed no difference between the two binders. D’Haese et al. [20] found that the number of patients with renal osteodystrophy decreased in the lanthanum group, whereas that in the CC group increased. Malluche et al. [21] found an improvement in bone turnover during the first year as well as a significant improvement in bone volume during the second year. By contrast, Spasovski et al. [22] found no significant differences in the osteoblast number, bone formation rate, osteoid volume, or mineral apposition rate in the lanthanum and CC groups after a one-year treatment. None of these trials found association with aluminium-like bone toxicity after treatment of lanthanum. A standard and uniform evaluation system for bone disorder in CKD-MBD must be established to improve the assessment of the effects of LC on ROD.

Sevelamer is another calcium- and aluminum-free phosphate binder. A small number of studies directly compared this binder with LC. Only two cross-over studies were identified, and our meta-analysis showed that the two treatments were similarly effective in controlling serum calcium and phosphorus levels. However, compared with LC, SH can improve the lipid profile by reducing the total cholesterol and LDL levels. SH differs from other phosphate binders because of its unique ability to reduce the levels of serum cholesterol and proinflammatory factors. However, it also increases the risks of hyperchloremic metabolic acidosis and hyperkalemia. Sevelamer binds to bile acids probably because of its physiochemical similarities to common bile sequestrants. This characteristic allows sevelamer to interfere with fat absorption and reduce LDL cholesterol levels [45]. In addition, sevelamer can physicochemically bind to the negatively charged lipid A portion of endotoxin (ET). In vitro experiments showed that SH can bind to ET in a dose-dependent manner [46]. Moreover, an in vivo experiment demonstrated that sevelamer can reduce ET which was triggered by renal failure [47]. Previous trials [48-50] showed that compared with calcium-containing phosphate binders, sevelamer reduces the levels ET and proinflammatory markers such as CRP, interlekin-6, endothelin-1, and plasminogen activator inhibitor-1 in dialysis patients. In patients with early diabetic CKD, sevelamer carbonate significantly reduces HbA1c, fibroblast growth factor 23, lipids, tumor necrosis factor-α, and oxidative stress compared with CC [51]. However, the studies included in our systematic review did not compare the anti-inflammatory effects of LC and SH. Compelling preliminary data demonstrate that the ET-binding effect and anti-inflammatory activity of SH are associated with the improvement of mortality in ESRD compared with those of calcium-containing phosphate binders [52]. Long-term clinical trials must be conducted to confirm the relationship between the amelioration of lipid metabolism and the improvement of patient survival. In particular, we recommend that additional studies be performed to determine if the lipid-lowering effect or anti-inflammatory activity of SH can improve the clinical outcome for CKD–MBD patients compared with LC.

The included studies did not compare the serum bicarbonate and potassium levels of the different groups. Moreover, the risks of acidosis and hyperkalemia of SH were unknown. Sevelamer carbonate is an improved, buffered form of SH that has equivalent efficacy in controlling phosphate levels but has a lower incidence of the above adverse effects [53]. Future RCTs that compare LC and sevelamer carbonate is also recommended.

The safety of LC has received considerable concern. All of the studies included in this paper reported that the accumulation of LC in both blood and bone was below toxic levels. After six years of LC treatment, the incidences of fractures and bone-related musculoskeletal adverse events were also significantly low [54]. In addition to that in bone, lanthanum accumulation in the liver should also be a point of concern because LC is excreted through bile. Although increased liver lanthanum deposition after oral lanthanum loading in uremic rats in comparison with normal renal function rats was observed [55,56], a subsequent investigation showed that lanthanum was present in the lysosomes of hepatocytes and was mostly concentrated in the biliary poles of the hepatocytes and within the bile canaliculi [57]. No lanthanum was detected in the hepatocyte mitochondria, nucleus, or cytoplasm. The six-year, long-term clinical observation also showed that liver enzymes did not increase, and that the few cases of liver- or biliary-related adverse events, none of which were considered to be related to lanthanum, were mainly observed in the first two years of treatment [54]. However, one case study reported that lathanum induced abnormal liver function in one male patient with PD and in one female patient with HD [58]. Our systematic review cannot provide sufficient evidence to show the safety of lanthanum in liver function. Therefore, future studies should also investigate the concentration and possible toxicity of lanthanum in the liver.

The most commonly reported side effects were gastrointestinal adverse events. Our meta-analysis showed no differences in nausea, constipation, and dyspepsia. The prevalence of vomiting was significantly higher in LC compared with that in CC. LC had lower risks of diarrhea and intradialytic hypotension compared with placebos and previous phosphate binders, respectively. Other side effects included dialysis complications, bronchitis, rhinitis, and pruritus while no significant differences were found between the treatments. One study showed that LC-treated patients had lower risks of intradialytic hypotension, diarrhea, cramps or myalgia, and abdominal pain compared with those treated with their previous phosphate binders.

Note that the effects of the dialysis type, dialysis dose, session duration, membrane type, and dialytic modality on phosphate removal were not assessed in the meta-analysis. Only four trials included patients with peritoneal dialysis. Therefore additional RCTs should be conducted to investigate the effects of lanthanum in peritoneal-dialysis patients.

To our knowledge, this study is the first to conduct a comprehensive systematic review of RCTs that assessed the advantages and disadvantages of using lanthanum treatment for CKD-MBD in dialysis patients. A previous [25] systematic review evaluated the effects of all phosphate binders on CKD-MBD in patients with CKD and also confirmed the efficacy of lanthanum in reducing the phosphorus levels (similar to that of CC) and lowering the incidence of hypercalcemia. However, the study did not evaluate the effects of lanthanum on mortality, vascular calcification, and bone disorder. Moreover, it did not provide the detailed side effects of lanthanum on bone, plasma, and liver contents nor include peritoneal-dialysis patients. Other published meta-analyses focused only on SH [59] or included both observational studies and RCTs [60].

Our systematic review has a number of limitations. Except for two trials with large sample sizes of 1359 [23] and 767 [31], most of the trials enrolled a limited number of patients. The duration of half of these trials ranged from 4 weeks to 8 weeks. The key findings are limited by the lack of long-term studies on mortality, cardiovascular events, cardiovascular calcification, and bone disorder. Most of the included trials only observed the biochemical parameters without considering patient-focused outcomes. Moreover, this review did not evaluate the health economic effectiveness of LC. Although LC can reduce the serum phosphorus level and has a lower risk of vascular calcification and hypercalcemia, it is a rare metal that is more valuable than calcium. A number of studies focused on the cost-effectiveness ratio of phosphate binders [61,62]. One study [61] showed that LC is cost-effective as a second-line treatment for patients who are not adequately maintained on CC (serum phosphorus above 5.6 mg/dL). Therefore, a systematic review that evaluates the health economic effectiveness of LC must be conducted to serve as a guideline for clinicians in providing individualized therapies for different patients, particularly for those in developing countries.

## Conclusions

The results of this meta-analysis show that lanthanum efficiently lowers the serum phosphorous and iPTH levels without elevating the serum calcium level. Apart from a higher incidence of vomiting, LC did not demonstrate higher incidence rates of other adverse effects compared with other treatments. Moreover, the accumulation of LC in blood and bone was below toxic levels. Current evidence is insufficient to demonstrate that lanthanum is superior to other phosphate binders in terms of lowering mortality, cardiovascular events, and vascular calcification as well as of improving bone disorder. Future studies with larger sample sizes and longer durations must be conducted to assess the effects of lanthanum on not only biochemical outcomes, but also on mortality, cardiovascular events, vascular calcification, and bone disorder. In addition, comparison with sevelamer carbonate, safety in bone and liver, health economic effectiveness, dialysis method and prescription should also be concerned in the future studies with LC.

## Abbreviations

CKD-MBD: Chronic kidney disease mineral and bone disorder; ESRD: End-stage renal disease; LC: Lanthanum carbonate; RCT: Randomized controlled trials; iPTH: Intact parathyroid hormone; CC: Calcium carbonate; SH: Sevelamer hydrochlorid; CV: Cardiovascular; HD: Hemodialysis; CAPD: Continuous ambulatory peritoneal dialysis; CENTRAL: Cochrane Central Register of Controlled Trials; CBBs: Calcium-based binders; NCBs: Non-calcium binders; CT: Computed tomography; TAP: Total alkaline phosphatase; BAP: Bone-specific alkaline phosphatase); RR: Risk ratios; CI: Confidence intervals; MD: Mean difference; SMD: Standardised mean difference; ROD: Renal osteodystrophy; DCOR: Dialysis Clinical Outcomes Revisited.

## Competing interests

The authors declared that they have no competing interests.

## Authors’ contributions

ZCL was involved in conducting the literature searches, obtained and analysed studies and wrote the manuscript of the paper. WJ was involved in conducting the literature searches, obtained and analysed studies. LZ was involved in the design of the study, adjudication on disagreements in data analysis, writing and editing of draft and final versions of the paper. FJM was involved in the design of the study. All authors have read and approved the final manuscript.

## Pre-publication history

The pre-publication history for this paper can be accessed here:

http://www.biomedcentral.com/1471-2369/14/226/prepub
